# Multi-stimuli responsive and multi-functional oligoaniline-modified vitrimers†
†Electronic supplementary information (ESI) available. See DOI: 10.1039/c6sc02855a


**DOI:** 10.1039/c6sc02855a

**Published:** 2016-09-05

**Authors:** Qiaomei Chen, Xiaowen Yu, Zhiqiang Pei, Yang Yang, Yen Wei, Yan Ji

**Affiliations:** a The Laboratory of Bioorganic Phosphorus Chemistry and Chemical Biology , Department of Chemistry , Tsinghua University , Beijing 100084 , China . Email: weiyen@tsinghua.edu.cn ; Email: jiyan@mail.tsinghua.edu.cn

## Abstract

Smart polymers have been playing indispensable roles in our lives. However, it is challenging to combine more than three stimuli-responses or functionalities into one polymer, not to mention integrating multi-stimuli responsivity and multi-functionality at the same time. Vitrimers, an emerging type of materials, are covalently crosslinked networks that can be reprocessed but are still infusible and insoluble. Herein, we show that simply introducing oligoaniline into a vitrimer results in a covalently crosslinked material that can respond to six different stimuli (heat, light, pH, voltage, metal ions and redox chemicals) and perform six functions (shape memory, welding, healing, recycling, electro-chromism and adsorption of metal ions). New properties, which cannot be found in either neat vitrimers or oligoanilines, are generated, including photo-heal-ability, photo-weldability, pH-induced shape memory, enhancement of the photo-thermal effect due to metal ions absorption and simultaneous multi-tasking operations. Furthermore, the material is low-cost and suitable for large-scale mass production.

## Abstract 2

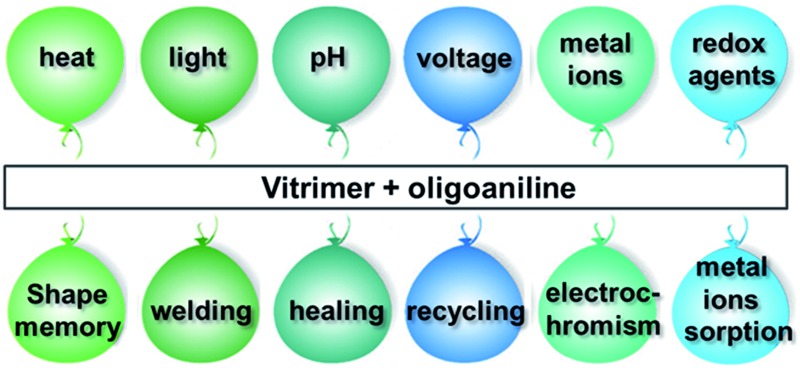
Introducing oligoaniline into a vitrimer resulted in a smart material that simultaneously responds to six different stimuli and performs six different functions.

## Introduction

Smart polymers are able to change shapes, dimensions, chemical or physical properties in real time in response to external stimuli (such as temperature,^1,2^ light,^3–5^ electric or magnetic fields,^6–8^ pH,^9,10^ moisture^11^ and chemicals^12^). Driven by the rapidly increasing demand for technological applications, such as actuators,^13–15^ sensors^16–18^ and controllable drug-delivery systems,^19–21^ they have gained tremendous attention. Over the last decade, researchers have started to create multi-stimuli responsive polymers (MSRPs) and multi-functional polymers (MFPs).^22–24^ Both are expected to enhance the versatility and capacity of materials in multifarious applications. Even though great progress has been made in both areas, they face a similar challenge: the number of stimuli or functionalities is normally limited to less than three. The few exceptions are all based on supramolecular chemistry or gels.^25–28^ Not only are these materials unable to be used as engineering materials, but they are also generally vulnerable to heat or solvents.

It remains very challenging to make a polymer with more than three functionalities or responsivities, that is to say, to make it respond to multiple responses and perform multiple functionalities at the same time. Currently, the most widely used strategy to construct MSRPs or MFPs is either incorporating special groups into polymers or using block copolymers. It is hard to design and combine more than three compatible moieties or blocks with different responsive behaviors or functionalities into the same polymer. Composites work when adding additional external stimuli or functionality; but normally, one type of filler can only respond to one signal or possess one function. Recently, interpenetrating networks have been utilized to make MFPs.^29^ However, only two networks with different functionalities can be integrated together. For thermosetting polymers, which are notorious for their difficulty in processing, to achieve both multi-responsivity and multi-functionality is even more challenging.

Vitrimers are covalently crosslinked polymer networks that can be reprocessed, but are still insoluble and infusible at elevated temperatures, differing from the behaviors of thermoplastics (malleable and soluble) and thermosets (unprocessable).^30^ The processability of vitrimers derives from associative exchange reactions,^31^ leading to the formation of new covalent bonds and breaking of old ones at the same time. Thus, the cross-linking density is fixed and the polymer will not depolymerize during processing. A number of vitrimers, based on exchange reactions such as transesterification,^32–34^ olefin metathesis,^35^ vinylogous transamination^36^ and disulfide rearrangements,^37,38^ have been reported. However, most work in this area has focused on the introduction of new exchangeable links to form new types of vitrimers. Only recently has the exploration of their applications been started. Using imine type vitrimers, Zhang's group has invented an ultra-thin solid-state Li-ion electrolyte membrane^39^ and fully recyclable carbon fiber composite.^40^ Based on transesterification, our group has shown that vitrimers are able to produce multi-shape memory effects and moldable liquid crystalline elastomer actuators.^41,42^ We have also shown that carbon nanotubes (CNTs) can be dispersed into vitrimers to make them not only heat-responsive, but also light-responsive.^43,44^ However, none of the previous works can make the material responsive to more than three stimuli, nor can they bring multi-stimuli responsivity and multi-functionality together.

Herein, we present a smart covalently crosslinked polymer that can respond to six different stimuli and perform various functions by introducing a small amount of aniline trimer (ACAT, an oligoaniline) into a vitrimer. The chemistry involved is very simple. Oligoanilines, compared with polyaniline, have a well-defined molecular structure, excellent electro-activity and good solubility in common solvents. It is well known that oligoanilines can change between different oxidation states due to their redox properties and be doped/un-doped by acid/base. In the past, incorporating oligoanilines into polymers was mainly to make the polymer electroactive or pH responsive.^45–49^ Other applications have been seldom explored. As polyaniline has shown a strong photo-thermal effect and metal ions absorption capability,^50,51^ we assume that those properties should also be available in oligoanilines. Indeed, both are verified in this work. Therefore, we can here show that the addition of oligoaniline makes a vitrimer, which is only thermal-responsive, become also responsive to light, pH, voltage, metal ions and redox chemicals. The intrinsic thermal related functions of the vitrimers (shape memory, welding and recyclability) are well preserved. Meanwhile, the material can be used for metal ions absorption and electrochromic materials. Furthermore, the combination of oligoaniline with vitrimer generates new functions that are impossible for either oligoaniline or neat vitrimer alone to perform. Those functions are: pH-induced shape memory, light manipulated activities (including shape memory, welding and healing), metal ion enhanced light controlled activation. To show the benefits from multi-stimuli responses and multi-functionality, some examples have also been demonstrated here.

## Results and discussion

### Preparation and characterization of the ACAT–vitrimer

**Fig. 1:**
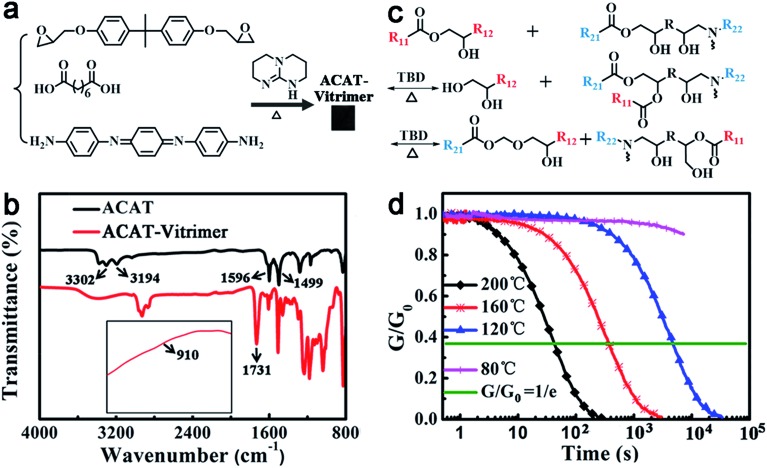
Polymer synthesis and characterization. (a) Synthesis of ACAT–vitrimer. (b) FTIR spectra of ACAT and ACAT–vitrimer. (c) An illustration of reversible transesterification. (d) Stress relaxation experiments of ACAT–vitrimer at different temperatures.

The oligoaniline containing vitrimer (ACAT–vitrimer) was prepared by curing diglycidyl ether of bisphenol A (DGEBA), suberic acid and ACAT in the presence of triazobicyclodecene (TBD) as the transesterification catalyst (Fig. 1a). It is known that the curing reaction of di-epoxy and di-carboxyl acid is a complicated process. Once the reaction between epoxy and acid begins, hydroxyl groups are generated. They can react with epoxy or carboxyl groups, leading to the formation of a network. In our case, ACAT also acts as a tetra-functional cross-linker to epoxy groups during the curing process. FTIR spectroscopy (Fig. 1b) confirmed the completion of the curing reaction. The characteristic peaks of epoxy (910 cm^–1^) and terminal amine (3302 cm^–1^ and 3194 cm^–1^) disappeared. At the same time, characteristic peaks of hydroxyl (3385 cm^–1^) and ester (1731 cm^–1^) increased. Swelling experiments (ESI, Fig. S2†) revealed that the obtained film maintained a network in trichlorobenzene at elevated temperatures (below the decomposition temperature). According to differential scanning calorimetry (DSC) (ESI, Fig. S3†), the glass transition temperature *T*_g_ of the resultant material was about 40 °C upon heating. As shown in Fig. S4,† a new transition (about 160 °C) occurred in the dilatometry test, which was defined by Leibler *et al.* ^52^ as *T*_v_ (topology-freezing transition temperature). The *T*_v_ of the ACAT–vitrimer is similar to that of the reported CNTs-dispersed vitrimer epoxies.^43^ Owing to the presence of transesterification catalyst TBD, ester groups are capable of exchanging with hydroxyl groups to generate new ester groups and new hydroxyl groups at temperatures sufficiently higher than *T*_v_ to trigger the exchange reaction (Fig. 1c). Therefore, vitrimers can be reprocessed at temperatures above *T*_v_.^30,31^ As shown in Fig. 1d, the relaxation time τ* (*i.e.*, the time needed for the sample to relax to *e* ^–1^ of its initial stress relaxation modulus) of the ACAT–vitrimer at 200 °C and 160 °C is 42 s and 373 s, respectively. However, τ* is much longer for the films relaxing at a temperature below *T*_v_ (*e.g.* 120 °C and 80 °C). This is because, at temperatures below *T*_v_, the exchange reaction is quite slow and the topology is frozen like the classical covalently crosslinked thermosets. What is intriguing, but reasonable, is that we also found that ACAT could also catalyze the transesterification reaction, even though the catalytic efficiency is much weaker than that of TBD (the details can be found in ESI, Fig. S6 and S7†).

### Properties similar to neat vitrimers

**Fig. 2:**
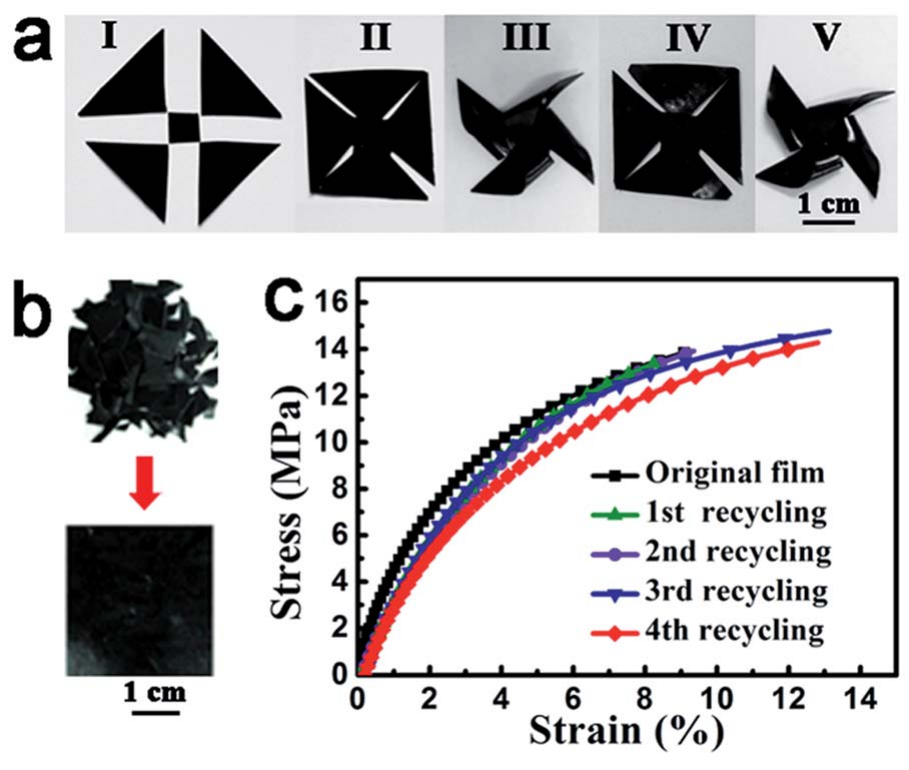
Heat responses of ACAT–vitrimer due to the intrinsic malleable property of vitrimers. (a) Welding (I–II) at temperature above *T*_v_ (200 °C, oven), permanent reshaping (II–III) at temperatures above *T*_v_ (200 °C, oven), temporary reshaping (III and IV) above *T*_g_ but below *T*_v_ (80 °C, oven) and shape memory (IV and V) by reheating above *T*_g_ (80 °C, oven). The thickness of the vitrimer films used here was about 0.2 mm. (b) Demonstration of the recycling process (with a thickness of about 0.2 mm). (c) Stress–strain curves for the recycled and original films.

Like vitrimers without ACAT (neat vitrimer), ACAT–vitrimer is heat-responsive. First of all, the obtained ACAT–vitrimer not only has an excellent shape memory effect,^53^ due to the existence of *T*_g_, but can also be welded and reshaped. In Fig. 2a, at 200 °C in the oven (*T* > *T*_v_), five pieces (I) were first welded into shape II and then reconfigured into a pinwheel-like permanent shape (III). The pinwheel was further reshaped into a flat temporary configuration IV at 80 °C. As expected, it changed quickly from shape IV to the permanent shape V, which is nearly the same as the shape III, at 80 °C in the oven. Secondly, ACAT–vitrimer shows recyclability. A new film was generated by cutting the film into pieces and then hot-pressing them under a pressure of 2 MPa for 10 min at 200 °C (Fig. 2b). The recycling experiment was repeated four times. Similar to neat vitrimers,^32^ there is only a slight decrease in the elastic modulus (Fig. 2c), which is probably due to the side reactions such as hydrolysis and alcoholysis of the ester groups at elevated temperature and pressure.

### Properties similar to ACAT

**Fig. 3:**
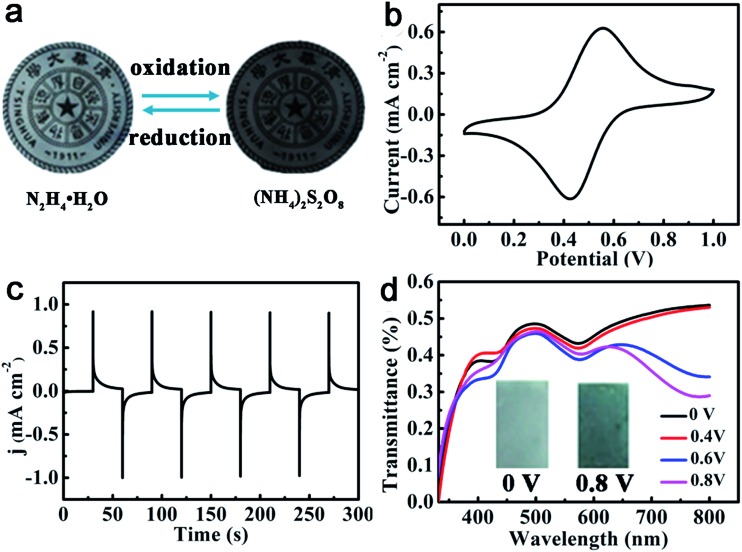
Responsive properties of ACAT–vitrimer to redox chemicals and voltages. (a) Chemical redox response of ACAT–vitrimer. (b) Cyclic voltammetry curve of ACAT–vitrimer on FTO conducting glass measured in 0.5 M H_2_SO_4_ (a solution of H_2_O and DMSO) at a scan rate of 50 mV s^–1^. (c) Chronoamperometry curves when the anodic potential was switched between 0 V and 0.8 V (*vs.* Ag/AgCl) for 30 s at each step. (d) Transmittance spectra of ACAT–vitrimer film under different applied potentials in the range of 0 V to 0.8 V (*vs.* Ag/AgCl) for 30 s at each potential (inset: photographs of ACAT–vitrimer at voltages of 0 V and 0.8 V).

It is known that oligoanilines can undergo reversible oxidation and reduction *via* both chemical and electrochemical processes.^54,55^ ACAT–vitrimer is also responsive to redox reagents and electric fields. In accordance with the reported ACAT-containing electroactive polymers,^56,57^ when a thin film was oxidized with the addition of an oxidant (ammonium persulfate) and reduced by a reductant (hydrazine hydrate), the transmittance of the film changed visibly (Fig. 3a) due to the transition of ACAT from the leucoemeraldine base (LB) to the emeraldine base (EB) in the film. The electric response of ACAT–vitrimer was characterized by a typical three-electrode electrochemical cell and UV-vis spectra. The cyclic voltammetry (CV) curve of ACAT–vitrimer (Fig. 3b) shows only one pair of redox peaks at 0.54 V and 0.45 V, which is assigned to the transition from LB to EB, and the values of the peaks are nearly identical with thermosetting epoxy containing ACAT.^45^ Fig. 3c shows 5 chronoamperometry cycles of the film, switching the voltage between 0 V and 0.8 V (*vs.* Ag/AgCl), which reveals that the current density has become constant within 30 s. Due to the electro-activity, ACAT–vitrimer has another functionality, which is electro-chromism. Upon changing the applied potentials from 0 V to 0.8 V, the color changed from light gray to dark blue (the inset of Fig. 3d). According to the UV-vis spectra of the film (Fig. 3d), the absorbance at 780 nm increased gradually, indicating the formation of an oxidized state for the film.^58^ Moreover, the total transmittance changed by about 25%.

ACAT–vitrimer can also absorb heavy metal ions from solutions. The ability of polyaniline to absorb heavy metal ions has been reported, which is due to the coordination interaction between amino groups of polyaniline and metal ions.^59,60^ For polymers containing oligoanilines, such a property was ignored in the past. The ACAT–vitrimer we report here also demonstrates the ability to extract heavy metal ions from solutions because of the amino and hydroxyl groups in the material. To investigate the adsorption ability of this material quantitatively, we cured the network on a solid support, silica gel, in one pot (the details can be found in the ESI†). Thermogravimetric analysis (TGA) showed that the mass percentage of polymer was about 31.1% (ESI, Fig. S8†). UV-vis spectra were utilized to determine the retention ability of copper ions (ESI, Fig. S9†). As shown in Fig. S9c,† saturation was reached in about 10 min and the maximum adsorption was about 0.10 mmol g^–1^ (details can be found in the ESI†). Although the maximum adsorption of ACAT–vitrimer was lower than that of the reported polyaniline,^59,61^ it still shows that ACAT–vitrimer can efficiently remove heavy metal ions from solutions. Moreover, it should be pointed out that, like polyaniline can absorb various metal ions,^60–66^ the metal ion absorption of ACAT–vitrimer is not limited to copper ions.

### Properties distinct from vitrimers and ACAT

#### Light responses due to the photo-thermal effect of ACAT

**Fig. 4:**
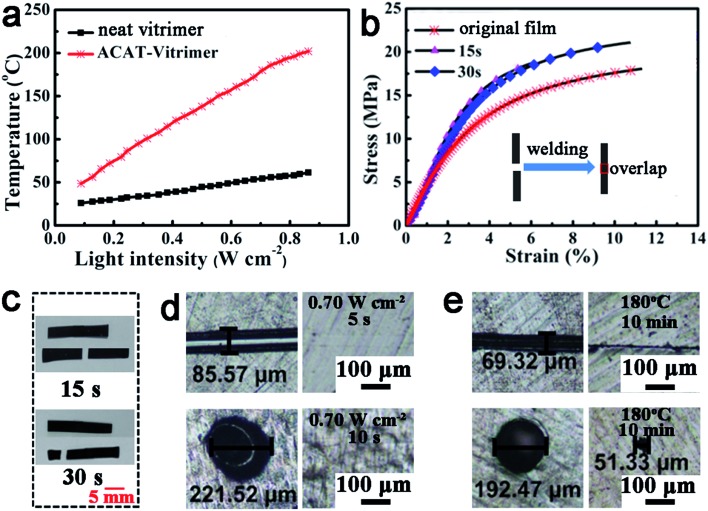
Photo-thermal effect enabled welding and healing of ACAT–vitrimer by light. (a) The temperature increase of ACAT–vitrimer and film without ACAT (neat vitrimer) upon light irradiation. (b) Stress–strain curves of the original film and films welded for 15 s and 30 s (with a thickness of about 0.2 mm) with a ramp rate of 0.4 N min^–1^ (inset: an illustration of the welding process. To obtain a robust connection, under light irradiation, the overlapping region was first manually compressed using a piece of quartz glass for 2 s and then the glass was removed, obtaining a pre-welded sample. Then the pre-welded film was directly exposed to IR light to get a robust connection). (c) Pictures of the welded films before and after stress–strain experiments (the thickness is about 0.2 mm). (d) Light-triggered (0.70 W cm^–2^) healing of ACAT–vitrimer with scratch (top) healed by local irradiation for 5 s (top right) and needle pierced hole (bottom) similarly healed within 10 s. (e) Control experiments using direct heating of scratch and pierced hole microscale defects (180 °C for 10 min) of ACAT–vitrimer.

We found that ACAT exhibited a photo-thermal effect. Polyaniline can absorb light and convert it into heat. It has been used as photo-thermal agents in various applications such as patterned functional layers,^67^ photo-thermal therapy^68^ and welding polymers.^69^ Surprisingly, the photo-thermal effect of oligoanilines has hitherto not been investigated. We measured the temperature of vitrimer with and without ACAT using an infrared thermal imager after the samples were exposed to near-infrared (IR) light (*λ* = 808 nm) for about 10 s to get an equilibrium temperature at different light intensities. As shown in Fig. 4a, the temperature of ACAT–vitrimer is much higher than that of neat vitrimer at each intensity. For example, at a light intensity of 0.70 W cm^–2^, the temperature of ACAT–vitrimer is about 180 °C while that of neat vitrimer is only about 54 °C.

As a result, light can be used to control the thermal related shape memory and welding. As shown in Fig. S10,† the temporarily spiraled ACAT–vitrimer and neat vitrimer (deformed above *T*_g_ but below *T*_v_) were exposed to IR light with a light intensity of 0.22 W cm^–2^ for 10 s. In accordance with our expectation, ACAT–vitrimer quickly recovered to the original length, but neat vitrimer retained the temporary shape. Individual films can be welded together *via* light irradiation. For example, irradiating on top of two overlapped films (with a thickness of 0.2 mm) for 30 seconds can result in a robust connection between the films. According to stress–strain tests (Fig. 4b), the sample welded for 15 s had a low elongation at break (about 5%), while a longer time (30 s) irradiation makes the elongation at break approach that of the original film. Meanwhile, the sample welded for 30 s broke at the bulk material instead of within the overlapping region. The one welded for 15 s broke within the overlapping region (Fig. 4c).

Microscale cracks can be healed by light, while direct heating was much less efficient in this regard. We carried out two healing experiments by direct heating and light irradiation, respectively. The scratch and hole healed completely *via* IR light (0.70 W cm^–2^, *T* = 175–180 °C) for 5 s and 10 s, respectively (Fig. 4d). On the contrary, the damages failed to be healed effectively by direct heating at 180 °C in oven for about 10 minutes (Fig. 4e). A similar phenomenon has been reported by Zhao's group.^4^ They supposed that the reason why samples could be healed by light instead of direct heating was because of thermal expansion. To verify this, we designed another experiment (details can be found in the ESI and Fig. S12†). It proves that thermal expansion widens the cracks, which makes it difficult for the polymer chains to contact with each other. Meanwhile, it indicates that light is a more flexible and effective stimulus than direct heating. We suppose that the mechanism of healing is the same as that of welding, which is the photo-thermal effect. As light is turned on, the temperature of the exposed area is increased high enough to activate transesterification. This is the same as the healing of CNTs-dispersed vitrimers,^43^ as well as the healing of polymers by the photo-thermal effect of gold nanoparticles used by others.^4^ To verify this, we measured the temperature increase of the cut film and found that the temperature of the exposed area quickly rose to about 182 °C in 10 s (light intensity: 0.70 W cm^–2^). Therefore, the transesterification reaction could lead to the flow of the polymer when light was on (ESI, Fig. S13†). All the above reveal that ACAT–vitrimer has an excellent photo-thermal effect that endows the material with functions such as light-induced shape recovery, light-triggered healing and welding; like the vitrimers with CNTs^44^ and gold nanoparticles (GNPs).^70^

#### Metal ions enhanced photo-thermal effect

**Fig. 5:**
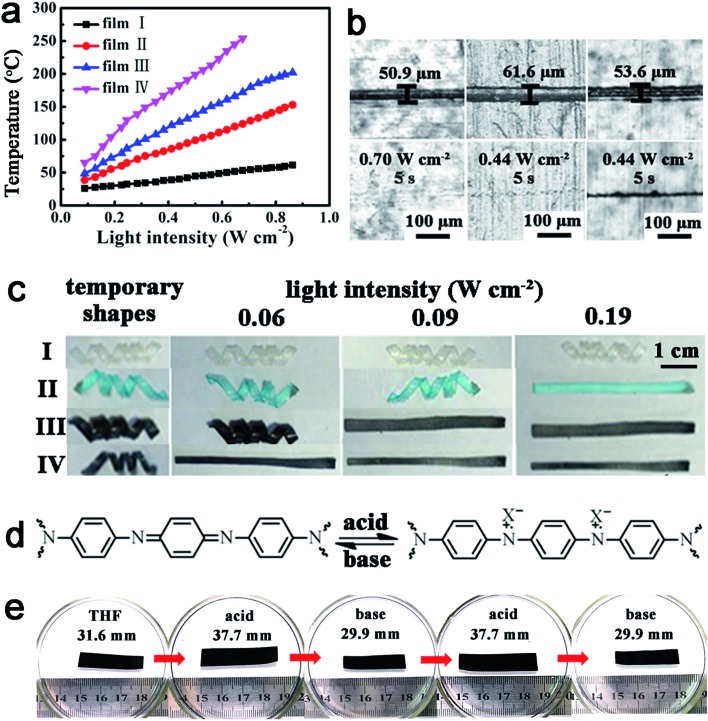
The effect of metal ions absorption on the light-triggered properties and the pH response. (a) The photo-thermal effect of the films I, II, III and IV (referring to neat vitrimer, neat vitrimer after absorbing Cu(ii), ACAT–vitrimer and ACAT–vitrimer after absorbing Cu(ii), respectively), where the content of ACAT was 1 mol% and the absorption experiments were conducted in 3 mmol L^–1^ copper(ii) acetate monohydrate/THF solution for 20 min. (b) The healing experiments of the films (from left to right, ACAT–vitrimer, ACAT–vitrimer swelled in 3 mmol L^–1^ copper(ii) acetate monohydrate/THF solution for 20 min and ACAT–vitrimer). (c) Shape recovery of films I, II, III and IV (the thickness of each film is about 0.2 mm) at different light intensities. (d) Reversible doping/un-doping of ACAT–vitrimer. (e) Extension–contraction response of an ACAT–vitrimer film (the thickness before swelling is about 0.2 mm) with changes in pH (acid and base solutions were 1 M PTSA/THF solution and 1 M TEA/THF solution, respectively).

We also found that the absorption of heavy metal ions can enhance the photo-thermal effect of ACAT–vitrimer. As shown in Fig. 5a, the temperature of films I, II, III and IV (referring to neat vitrimer, neat vitrimer after absorbing Cu(ii), ACAT–vitrimer and ACAT–vitrimer after absorbing Cu(ii), respectively) was measured using the infrared thermal imager at different light intensities (*λ* = 808 nm). It shows that the temperature of films II and IV increases more quickly than their control films I and III. The shape memory and healing experiments were also conducted to further demonstrate the enhancement of the photo-thermal effect. As shown in Fig. 5b, the ACAT–vitrimer absorbed Cu(ii) can be healed at a lower light intensity, but the control sample can only be healed at a higher light intensity. Owning to the different photo-thermal effect, the deformed films I, II and III recovered to their permanent shapes step by step at different light intensities (Fig. 5c). The neat vitrimer film IV showed no recovery, even at a relatively high light intensity. This experiment further justified the enhancement of the photo-thermal effect by metal ions.

#### PH-induced shape memory

As is known, polyaniline and its oligomers can be doped in acid and un-doped in base (Fig. 5d). We found that this allows a new shape memory effect due to a mechanism different from the heat-controlled shape memory due to *T*_g_.^71^ Doping and un-doping can cause the swelled ACAT–vitrimer to reversibly expand and contract, which has not been noticed in any oligoaniline containing polymers previously reported. The reported pH-responsive property of oligoanilines was mainly focused on regulating the self-assembly behaviors of oligoanilines and polymers containing oligoanilines:^48,72^ protonation/deprotonation leads to molecular structure changes. But here, we can use the protonation/deprotonation for shape memory. Fig. 5e demonstrates that the rectangular film, which has reached swelling equilibrium in tetrahydrofuran (THF), has the capacity to reversibly expand and contract when successively placed in acid (1 M *p*-toluenesulfonic acid, PTSA) and base (1 M triethylamine, TEA). From THF to acid, the length change was about 19%; and from acid to base, the length change was about 21%. Since the transesterification catalyst (TBD) is a stronger base than TEA, in contrast to ACAT, TBD would probably not be fully deprotonated and some of its vitrimer properties would be lost (for ACAT still maintain its catalytic effect). We verified this by a recycling experiment of the film treated with acid, then followed with base (details can be found in the ESI, Fig. S18†). Therefore, the recycled material is not exactly the same as the original ACAT–vitrimer in this case. This is similar to the material after absorption of metal ions. With metal inside, the chemical structure is changed. As shown in Fig. S14–S17,† copper ion absorption lowers the *T*_g_ and the transesterification reaction rate of ACAT–vitrimer, especially when the ACAT content is higher (details can be found in the ESI†). To recycle this material, it should be treated with an acid, then followed with a base. But the treatment would also lead to some change of the chemical structure. Therefore, strictly speaking, this material may not be fully recyclable in all situations.

In fact, without TBD as the catalyst, the non-vitrimer type epoxy with ACAT also possesses some stimuli-responsive properties similar to the ACAT–vitrimer. For instance, the non-vitrimer with 10 mol% ACAT also has an excellent pH-responsive property (details can be found in the ESI, Fig. S19†). Such a material can also revert to the permanent shape when heated or irradiated by light (ESI, Fig. S11†). Moreover, this material exhibited a notable reprocessing ability as we previously mentioned. But the ACAT–vitrimer with TBD as catalyst has better welding, healing and recycling performances. One can further increase the content of ACAT to improve the processing ability, but the mechanical property of the material will change. In terms of practical applications, where good processing is necessary, the ACAT–vitrimer used throughout this paper is a better choice.

#### Benefits from multi-stimuli responsivity and multi-functionality

**Fig. 6:**
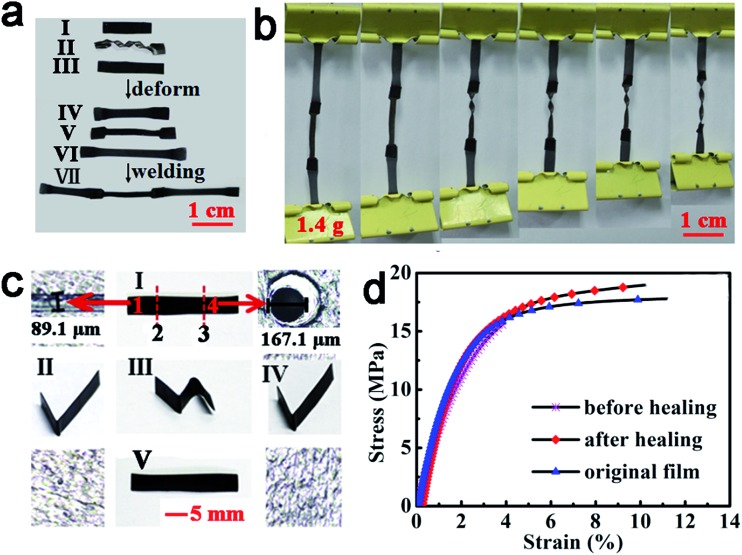
Manipulation of light-triggered properties on the same ACAT–vitrimer construct. (a and b) Successive welding and shape memory controlled by light (the thickness of each film is about 0.2 mm before deformation). (c) Sequential manipulation of healing and shape memory on the same film (with a thickness of about 0.2 mm) by light. (d) Stress–strain curves of the original film, damaged film and healed film with a ramp rate of 0.4 N min^–1^.

The combination of multi-stimuli responsivity and multi-functionality offers a high level of versatility in satisfying demanding requirements for practical applications. For a simple example, dual or triple optically triggered functionalities can be individually and non-interferingly operated on the same construct, which is impossible by direct heating. Since light can be controlled remotely, locally and temporarily, all the light controlled processes can be carried out at any time on the desired locations without affecting the surrounding areas, which is especially important when using this material in devices containing sensitive components or with complicated geometric shapes. As an illustration, photo-induced welding, reshaping and shape memory can be combined together to allow for flexible programming and doing mechanical work. As shown in Fig. 6a, films IV and VI were obtained by stretching films I and III with a strain of 100%, respectively. Film II was a permanent shape that was deformed by spiraling a flat rectangular film with IR light (0.70 W cm^–2^, *T* = 175–180 °C), from which the temporary shape V was fixed *via* IR light (0.22 W cm^–2^, *T* = 75–80 °C). Then, the films IV, V and VI were welded together by IR light (0.70 W cm^–2^) in 30 s. As the light was focused on the joint part, the temporary shapes of each segment were kept (if it were direct heating, the temporary shapes would be lost), resulting in the film shown in Fig. 6a(VII). Upon individually exposed each segment to light (0.22 W cm^–2^), all the segments recovered their permanent shapes successively (Fig. 6b). As the film was loaded with a weight of 1.4 g during the shape recovery process, a stress of about 78 kPa was generated. If proper programming is used, stress in different directions can be produced. Similarly, healing and shape memory of the same material can be realized. A scratch and a hole were firstly created on the film, as shown in Fig. 6c(I). Then, the film was folded into a temporary “N” shape (Fig. 6c(III)) upon the IR light exposure (0.22 W cm^–2^, *T* = 75–80 °C). The scratch was healed by local IR irradiation (0.70 W cm^–2^, *T* = 175–180 °C) for 5 s, but the sample remained almost the same shape. Upon exposing the right bend to light (0.22 W cm^–2^), the right part became flat (Fig. 6c(IV)). At last, the hole was healed (0.70 W cm^–2^) and the film recovered to the permanent shape (0.22 W cm^–2^), as shown in Fig. 6c(V). This test demonstrated that healing and shape recovery can be executed individually, which means that this material can be healed *in situ*. The stress–strain test reveals the mechanical property of the healed film is almost the same as the original (Fig. 6d). If the damaged sample was not healed, it would snap quickly with an elongation at break less than 4%.

**Fig. 7:**
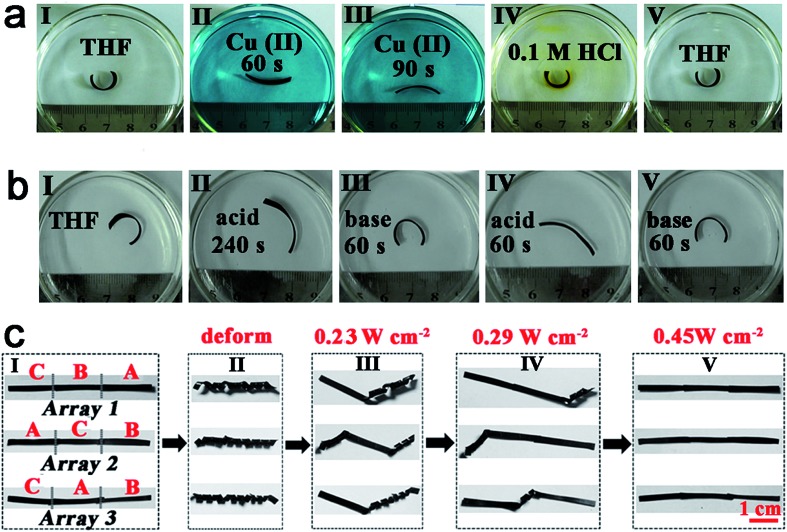
New shape memory performances. (a) Shape change of a bilayer responsive to metal ions and acid. (b) Shape change of the bilayer in different pH. (c) The multiple shape memory construct by welding films A, B and C (representative of ACAT–vitrimer, ACAT–vitrimer swelled in 1 mmol L^–1^ Cu(ii)/THF solution for 20 min and ACAT–vitrimer swelled in 3 mmol L^–1^ Cu(ii)/THF solution for 20 min) together. The thickness of each film is about 0.2 mm before deforming and the irradiation time of the shape recovery process at different light intensities was 10 s.

For a second example, welding by heat, pH-induced shape memory and metal ions absorption can work separately or together to make actuators. As the films are vitrimers, an asymmetric bilayer structure (BL, with a thickness of about 0.2 mm) can be constructed without any glue through simply welding ACAT–vitrimer (with a thickness of about 0.12 mm) with neat vitrimer (with a thickness of about 0.12 mm). Due to the different composition of each side of the BL, each side shows a different equilibrium swelling ratio in tetrahydrofuran (THF). The equilibrium swelling ratios of ACAT–vitrimer and neat vitrimer films were 137% and 160% in length, respectively (ESI, Fig. S20†). Therefore, the BL bends towards to the ACAT–vitrimer side after swelling in THF. As shown in Fig. 7a, the well-swelled film in THF was placed in 5 mmol L^–1^ copper(ii) acetate monohydrate/THF solution. The film bent to the opposite side within 90 s, which not only revealed the excellent ability of the material to extract metal ions, but also proved that the introduction of ACAT has enhanced the adsorption ability of neat vitrimer. Then, the film was moved into 0.1 M HCl, causing a destruction of the coordination interaction. Replacing the film in THF recovered the original shape. Furthermore, since ACAT–vitrimer could be doped with acid and un-doped with base, the aforementioned bilayer film could also change shape *via* pH differences. Upon doping with protonic acid, extension occurs to the side of ACAT–vitrimer, while the other side remains nearly unchanged. The asymmetric change induces the film to bend towards the side without ACAT in acid. The pH response experiments of BL films are shown in Fig. 7b. The well-swelled BL film (original shape) was firstly removed from THF to 1 M PTSA/THF solution. As the protonic acid doping started, the radius of the bent film gradually increased to form a new shape after 240 s. Then, the film was placed in 1 M TEA/THF solution. The film quickly recovered to the original shape in just 60 s. Then, the film was re-doped in acid within 60 s (details can be found in Fig. S21†). The aforementioned neutral-acid–base stimuli responsive experiments were repeated 5 times and showed excellent reproducibility.

For a third example, using photo-welding, light controlled shape memory and metal ions absorption enhanced photo-thermal effects, we can obtain a photo-responsive multiple shape memory construct. Multiple shape memory materials are capable of transforming from two or more programmed temporary shapes to its original shape step by step,^73^ which will greatly expand the practical applications of the shape memory materials. As a demonstration, films A, B and C (representative of ACAT–vitrimer, ACAT–vitrimer swelled in 1 mmol L^–1^ Cu(ii)/THF solution for 20 min and ACAT–vitrimer swelled in 3 mmol L^–1^ Cu(ii)/THF solution for 20 min) were welded together by light according to difference sequences (array 1, array 2 and array 3) (Fig. 7c(I)). As we have disclosed before, the photo-thermal efficiency of samples with different metal ion concentrations is different. And the photo-thermal effect of the samples in a cold environment, about –20 °C (placed in liquid nitrogen vapor, ESI, Fig. S22†) is still excellent. For example, the temperature of films A, B and C are 24 °C, 35 °C and 45 °C, respectively, when exposed to light with an intensity of 0.23 W cm^–2^ (ESI, Fig. S23†). All three constructs (array 1, array 2 and array 3) were deformed into shape II (Fig. 7c(II)). Exposing the constructs to light with an intensity of 0.23 W cm^–2^, only segment C recovered to its permanent shape (Fig. 7c(III)). Increasing the light intensity to 0.29 W cm^–2^, segment B recovered (Fig. 7c(IV)). At last, at an intensity of 0.45 W cm^–2^, segment A became flat (Fig. 7c(V)). Clearly, from left to the right, different arrays showed different recovery processes due to different connection sequences of films A, B and C. This enables a more flexible control for multiple shape memory performance.

As a demonstration of the ACAT–vitrimer being used in practical applications, we show here that it can be used as a wire insulation coat. As shown in Fig. S24,† a copper wire is closely wrapped by a square of ACAT–vitrimer film (I) and the edges are sealed by heat-welding at 200 °C for 10 min in an oven (II). When necessary, this wire can be deformed into a new permanent configuration. For example, the wrapped wire is further deformed into shape III and exposed to light (0.70 W cm^–2^) for 1 min to release the induced stress. As a result, its new permanent shape is fixed. For the insulation coat, cracks can be dangerous. As shown in Fig. S24(IV),† in the presence of a crack, we measured resistance between the crack and the end of the wire using a digital multimeter. The resistance was 0.8 Ω, which reveals that the crack has caused an electrical leak in the wire. Here, as the insulation is ACAT–vitrimer, big cracks of the insulation coat can be easily mended by welding (V). After mending, the wrapped wire can be straightened again (VI). Similarly, the microscale cracks can be optically healed in suit (VI, 0.70 W cm^–2^). Moreover, the ACAT–vitrimer insulation coat can be conveniently peeled off from the wire to recycle the polymer and the copper wire (VII). The recycled ACAT–vitrimer (VIII, made by hot-pressing the ACAT–vitrimer peeled off the copper wire) still has excellent shape memory effect. As shown in Fig. S24(IX),† the recycled film was stretched at 80 °C, then cooled to fix a temporary shape. When heated at 80 °C again, it quickly recovered to the permanent shape.

## Conclusions

In summary, using very simple chemistry and without sophisticated molecular design, we have successfully produced a smart polymer that combines multi-stimuli responsivity with multi-functional properties in the same material. As an emerging class of polymers, vitrimers have attracted a lot of attention for their unique properties as well their potential applications. The inherent properties of oligoaniline offer a powerful platform to greatly and easily expand the application of vitrimers. Neat vitrimer is heat-responsive. The addition of oligoaniline makes the vitrimer also responsive to light, pH, voltage, metal ions and redox chemicals. The properties of the obtained material are not a simple superposition of ACAT and vitrimer. The malleability of vitrimers as well as the metal ions absorption and electro-chromism of oligoanilines are well retained. Moreover, the combination of oligoaniline with vitrimer generates new functions that are impossible for either oligoaniline or neat vitrimer alone to perform. Those functions are: light-manipulated activities (including shape memory, welding and healing), pH-induced shape memory and metal ions absorption enhanced light-controlled activation. Although the fundamental impetus of the photo-thermal effect is thermal, microscale cracks can be healed more efficiently with light irradiation than direct heating. Healing is not observed in neat vitrimer, but when exposed to light, micro-cracks disappear. To show the benefits from multi-stimuli response and multi-functionality, some examples (simultaneous multiple task operations, pH or metal ions induced shape changes and multiple shape memory) were also demonstrated. Not only is this kind of material versatile, but it is also low-cost and suitable for large scale mass production. Moreover, the strategy induced here is not limited to the current system. It is possible to improve or optimize the responses and functionalities using other oligoanilines or vitrimers.
