# Supplementary material for: Multi-stimuli responsive and multi-functional oligoaniline-modified vitrimers
Source: Chem Sci. 2016 Sep 5;8(1):724–33. doi: 10.1039/c6sc02855a (PMC5458722; doi:10.1039/c6sc02855a)
Supplement: Supplementary file 1 [file SC-008-C6SC02855A-s001.pdf]

Electronic Supplementary Material (ESI) for Chemical Science.
This journal is © The Royal Society of Chemistry 2016

Electronic Supplementary Information

Multi-stimuli Responsive and Multi-functional Oligoaniline Modified Vitrimers

Qiaomei Chen, Xiaowen Yu, Zhiqiang Pei, Yang Yang, Yen Wei* and Yan Ji*

The Key Laboratory of Bioorganic Phosphorus Chemistry & Chemical Biology, Department of Chemistry, Tsinghua University, Beijing 100084, China.
E-mail: jiyan@mail.tsinghua.edu.cn, weiyen@tsinghua.edu.cn

1. Method

Synthesis of amino-capped aniline trimer (ACAT)

ACAT was synthesized by oxidative coupling of stoichiometric amount of bis (4-aminophenyl) amine sulfate hydrate and aniline with ammonium persulfate as the oxidant in 1 M HCl solution, according to *Macromolecules* 1998, **31**, 2702-2704.

Preparation of ACAT-Vitrimer film

ACAT-Vitrimer was prepared by reacting diglycidyl ether of bisphenol A (DGEBA), suberic acid and ACAT in the presence of triazobicyclodecene (TBD) as the transesterification catalyst. TBD was 5 mol% to the carboxyl groups. For the experiments related to heat and light responsivity, the concentration of ACAT is 1 mol%. All the heat and light responses of 1 mol % ACAT samples can be repeated on the samples with 10 mol% ACAT. But their responses to other stimuli are not as good as 10 mol% samples. Therefore, for other experiments, the concentration is 10 mol%.

To get a sample with 1 mol% ACAT, suberic acid and ACAT were 99:1 in molar ratio, and the total degree of functionality of epoxy groups and curing groups was 1:1. For example, DGEBA (1.01 mmol) and ACAT (0.01 mmol) were added into a 100 ml round-bottom flask. A suitable amount of N, N-dimethylformamide (DMF) was added to dissolve the chemicals and then the mixture was ultrasonically dispersed for 3 minutes. The reaction proceeded for 1 h at 100ºC, then stoichiometric ratios of suberic acid (0.99 mmol) and triazobicyclodecene (0.1 mmol) were added to react for another 1h. The pre-crosslinking mixture was transferred to a watch glass covered with teflon film and the solvent was evaporated at 120ºC while manually stirred. When most of the solvent had gone, the mixture was slowly warmed up to 160ºC while the stirring was continued until the system was too viscous to flow. Then the mixture was transferred into a mold and cured at 180ºC for 4 h under a pressure of 5 MPa.

ACAT-Vitrimer containing 10 mol% ACAT was synthesized by the similar process.

Preparation of neat vitrimer film

Neat vitrimer was prepared by directly stirring the stoichiometric amount of DGEBA (1 mmol), suberic acid (1 mmol) and TBD (0.1 mmol) in a watch glass covered with teflon film at 160ºC. When the mixture was too viscous to flow, it was transferred into a mold and cured at 180ºC for 4 h under a pressure of 5 MPa.

Preparation of non-vitrimer type epoxy film

The Preparation of the non-vitrimer type epoxy (with ACAT but without TBD) film is similar to the processes of ACAT-Vitrimer film. The main difference is that the non-vitrimer film does not contain the transesterification catalyst TBD.

Electrochemical experiments of ACAT-Vitrimer

The electrochemical measurements were operated on CHI 760D potentiostatgalvanostat (CH Instruments Inc.) using typically three-electrode electrochemical cell. The cyclic voltammetry data was measured in a 0.5 M H_2SO_4 mixture solution of dimethyl sulfoxide (DMSO) and deionized water with a scan range of 0 V to 1 V and scan rate of 50 mV s^{-1}. A platinum electrode and a saturated calomel electrode acted as the counter electrode and the reference electrode, respectively. The Vol. (1M H_2SO_4)/Vol. (DMSO) ratio was 1:1. The working electrode was obtained by spin-casting the aforementioned pre-crosslinking ACAT-Vitrimer mixture on the fluorine doped tin oxide (FTO) conductive glass with the spinning rate of 500 r min^{-1} for 15 s and 1000 r min^{-1} for 30 s then curing 2 h at 120ºC, 1 h at 140ºC and 0.5 h at 160ºC.

2. Characterization of amino-capped aniline trimer (ACAT)

To confirm the chemical structure of ACAT, liquid phase ^1H nuclear magnetic resonance (^1H NMR) spectroscopy was performed on a JEOL-ECX400 spectrometer. ACAT was dissolved in deuterated dimethyl sulfoxide (10 mg ml^{-1}), and the data were acquired at 25ºC. Signals appeared at 5.4 ppm is attributed to the terminal amine protons ($–NH_2$) of ACAT, and the rest characteristic peaks at around 7.0–6.5 ppm are in accordance with the aromatic proton of ACAT (Fig. S1a). The attenuated total reflectance FTIR spectra were conducted on a Perkin Elemer spectrum 100 at room temperature ranged from 4000 to 650 cm^{-1}. The characteristic absorption peaks at 3302 cm^{-1} and 3194 cm^{-1} are resulted from the terminal $–NH_2$. The characteristic absorption bands located at 1596 cm^{-1} and 1499 cm^{-1} belong to the vibrational bands of quinoid rings and benzenoid rings, respectively (Fig. S1b). The mass spectrum was run on a Shimadzu LCMS-IT-TOF high resolution mass spectrometry (HRMS). From Fig. S1c, we can see a molecular ion peak at 289.1443, which is corresponding to the molecular weight of ACAT.

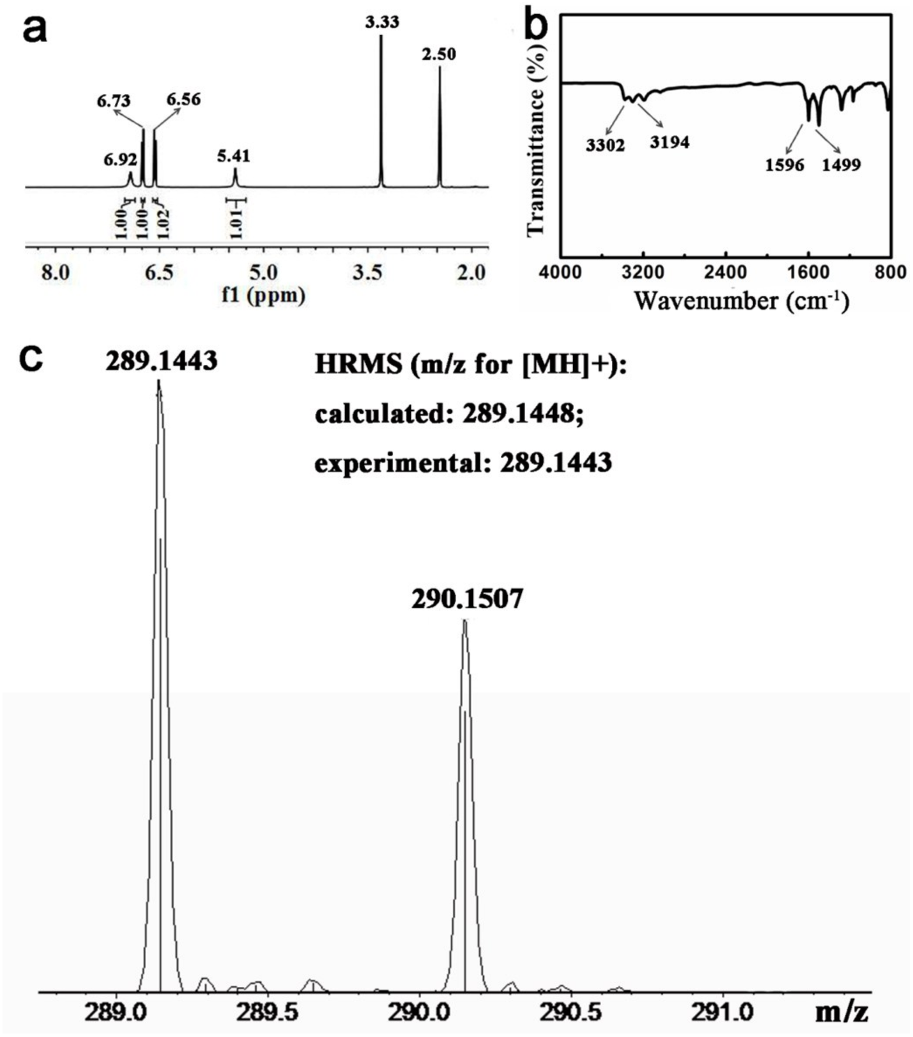

Fig. S1 Characterization of ACAT. (a) The ^{1}H NMR spectrum of ACAT. (b) The FTIR spectrum of ACAT. (c) The high resolution mass spectrum (HRMS) of ACAT.

3. Characterization of ACAT-Vitrimer

The swelling experiments were done using tricholorbenzene as the solvent. The volume of the film increased by 61% after 1 h at 100ºC and increased slowly after swelling 1 h at 140ºC, 180ºC and 20 h at 180ºC, resulting in a final volume increase of 115%. (Fig. S2)

The thermal property was measured on a differential scanning calorimetry (DSC) instrument TA Q2000 in conjunction with a controller and associated software to make up a thermal analysis system. The measure procedure consisted of two scanning cycle, where both the heating and cooling rate were 10ºC min^{-1}, and the first scanning was in order to eliminate the thermal history of the sample. As is shown in Fig. S3, the glass transition T_g is about 40ºC (the midpoint of the transition) upon heating.

The mechanical properties were studied with a TA Q800 DMA instrument. In the stress-strain experiments at 25ºC, the force ramping rate was 0.4 N min^{-1}. Dilatometry test was performed under a constant load of 10 kPa in a temperature ramping rate of 3ºC min^{-1} to 250ºC. T_v is calculated to be about 160ºC (Fig. S4).

Optical microscopy experiments were tested on a Nikon Eclipse LV100POL polarized light optical microscope, equipped with a Nikon Digital Sight DS-U3 digital camera and hot-stage.

The thermal stability of ACAT-Vitrimer was measured on a TA Q50 TGA under air and nitrogen atmosphere. (Fig. S5)

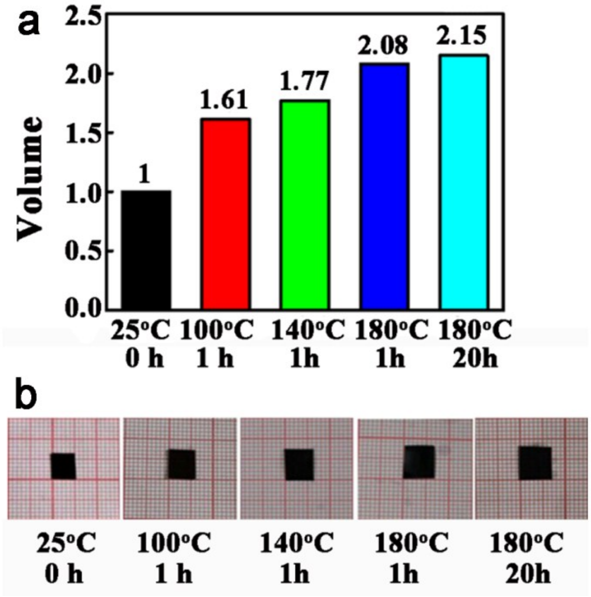

Fig. S2 Swelling experiments of ACAT-Vitrimer. (a) Volume changes of ACAT-Vitrimer film swelled in tricholorbenzene at different temperature. (b) Photographs of ACAT-Vitrimer film after swelled in tricholorbenzene at different temperature.

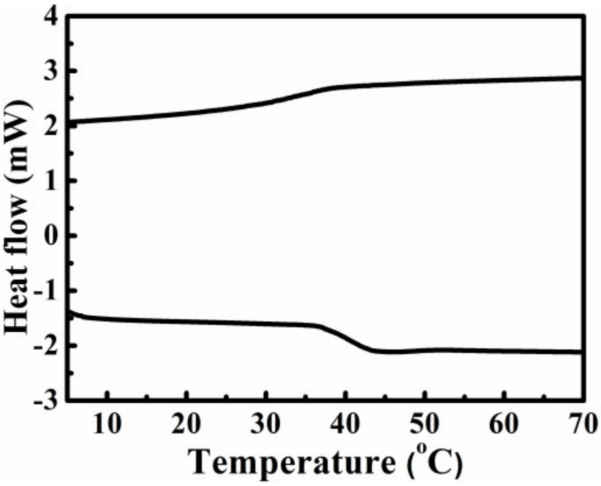

Fig. S3 DSC curve of both the heating and cooling procedures (rate of 10ºC min^{-1}).

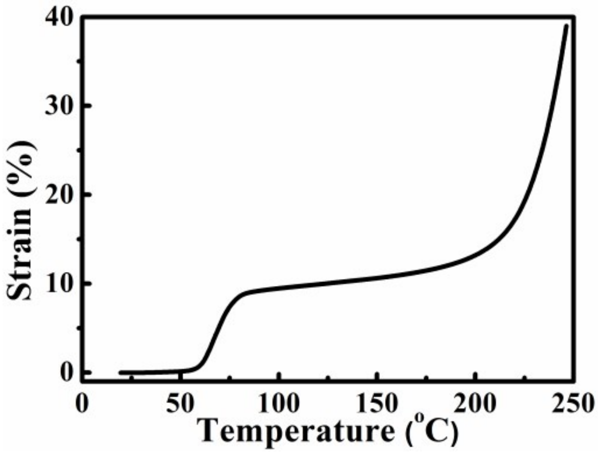

Fig. S4 Dilatometry test of ACAT-Vitrimer with a ramp rate of 3ºC min^{-1}.

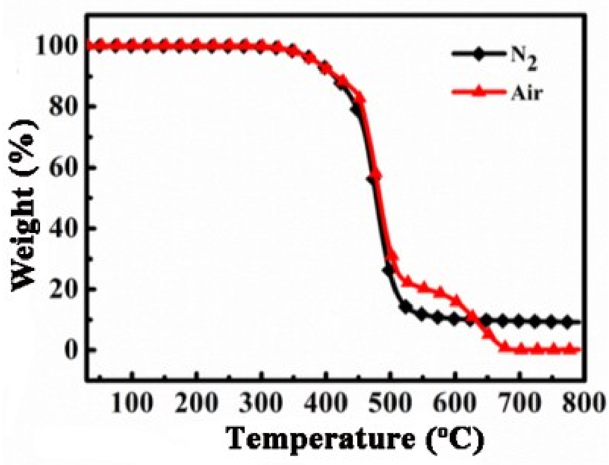

Fig. S5 TGA curves of ACAT-Vitrimer (heating rate: 20ºC min^{-1}).

4. Investigation on the catalytic effect of ACAT

To examine whether the transesterification reaction can be catalyzed by oligoaniline, we conducted the stress relaxation experiments on different samples. As is shown in the stress relaxation experiments conducted at 180ºC (Fig. S6), the stress of the non-vitrimer type epoxy without ACAT only relax about 20% after 10^4 s, which is probably caused by the side reactions such as hydrolysis and alcoholysis of the ester groups at elevated temperature and pressure (*Science*, 2011, **334**, 965-968). The relaxation time τ* of ACAT-Vitrimer with 1 mol% ACAT and ACAT-Vitrimer with 10 mol% ACAT is about 415 s and 724 s, respectively. The relaxation time τ* of the non-vitrimer type epoxy with 1 mol% ACAT and non-vitrimer type epoxy with 10 mol% ACAT is 4744 s and 3926 s, respectively. The molar ratio of TBD was 10 mol% to suberic acid, which was close to the molar ratio of ACAT (11 mol% to suberic acid) in non-vitrimer with 10 mol% ACAT. Therefore, ACAT indeed can catalyze the transesterification reaction, but the catalytic efficiency is much weaker than that of TBD.

We also conducted the recycling experiments to qualitatively justify the catalytic effect of ACAT. As is shown in Fig. S7, the non-vitrimer type epoxy without ACAT was hot-pressed for 30

min at 200ºC with a pressure of 5 MPa. The pieces can not be joined together. On the contrary, pieces of ACAT-Vitrimer with 1 mol% ACAT and ACAT-Vitrimer with 10 mol% ACAT could be easily reprocessed into intact films within only 10 min. Pieces of the non-vitrimer type epoxy with 1 mol% ACAT and non-vitrimer type epoxy with 10 mol% ACAT could also be combined together by hot-pressing but the surface of the recycled films are rough and has many visible cracks. The cracks on the recycled non-vitrimer type epoxy with 1 mol% ACAT are not only more but also larger than that of sample with 10 mol% ACAT, which means that the more ACAT, the better reprocessability.

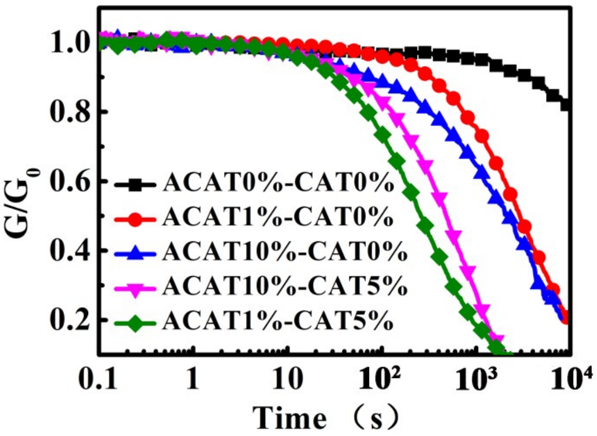

Fig. S6 The stress relaxation of different samples conducted at 180 ºC.

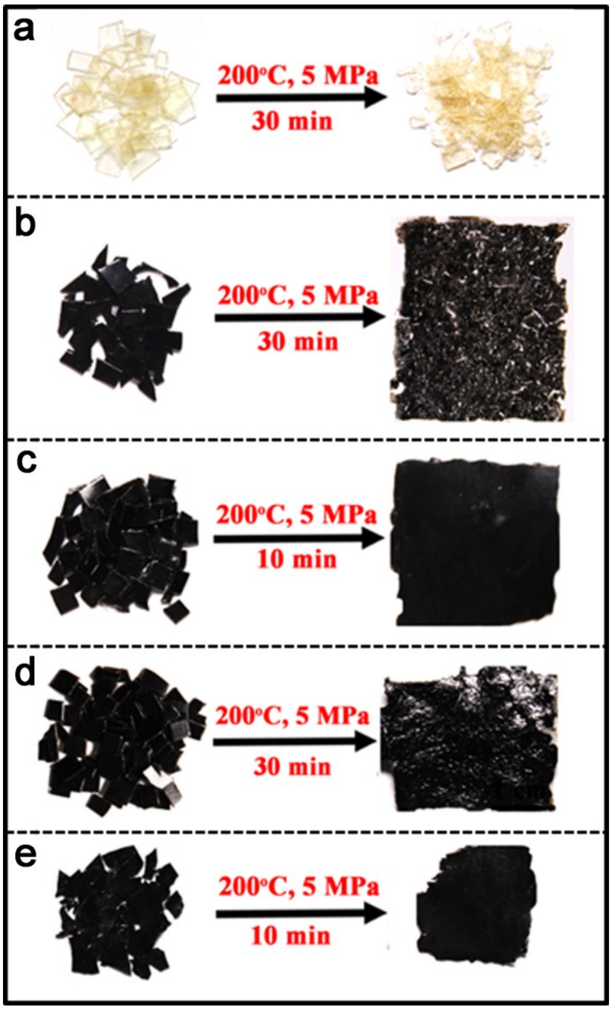

Fig. S7 Recycling experiments of different samples. (a) Non-vitrimer type epoxy without ACAT. (b) Non-vitrimer type epoxy with 1 mol% ACAT. (c) ACAT-Vitrimer with 1 mol% ACAT. (d) Non-vitrimer type epoxy with 10 mol% ACAT. (e) ACAT-Vitrimer with 10 mol% ACAT.

5. TGA curve of the polymer coated silica gel

The polymer coated silica gel was obtained by curing the network on the solid support silica gel in one pot using the aforementioned method, where the mass ratio of polymer and silica gel was 1:2. As shown in Fig. S8, the mass percentage of polymer was 31.1%.

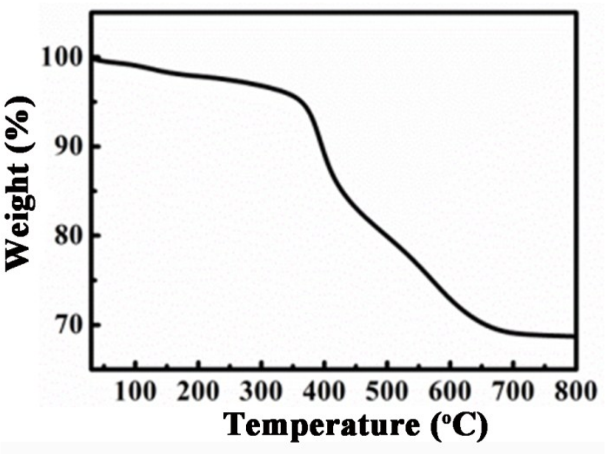

Fig. S8 TGA curve of the polymer coated silica gel in air (heating rate: 20ºC min^{-1}).

6. The quantitative retention ability of ACAT-Vitrimer for copper ions

UV-Vis spectra were utilized to determine the retention ability of copper ions. The UV-Vis adsorption spectra of the copper (**II**) acetate monohydrate/THF solutions showed a characteristic peak at 674 nm and their intensities increased with the copper ions concentration linearly (Fig. S9a), based on which the standard curve was established (Fig. S9b). The polymer coated silica gel (0.5 g) was introduced to 10 ml of the prepared 5 mmol L^{-1} Cu (**II**)/THF solution. The mixture was stirred at 650 r min^{-1} at 25ºC for different time ranging from 2 min to 20 min. Then the mixture was filter and the filtrate was analysed with UV-Vis spectra. The numbers of moles adsorbed (N_f) per gram of polymer was calculated from the standard curve. The results showed that the saturation reached about just 10 min and the maximum adsorption N_f was 0.10 mmol g^{-1} (Fig. S9c).

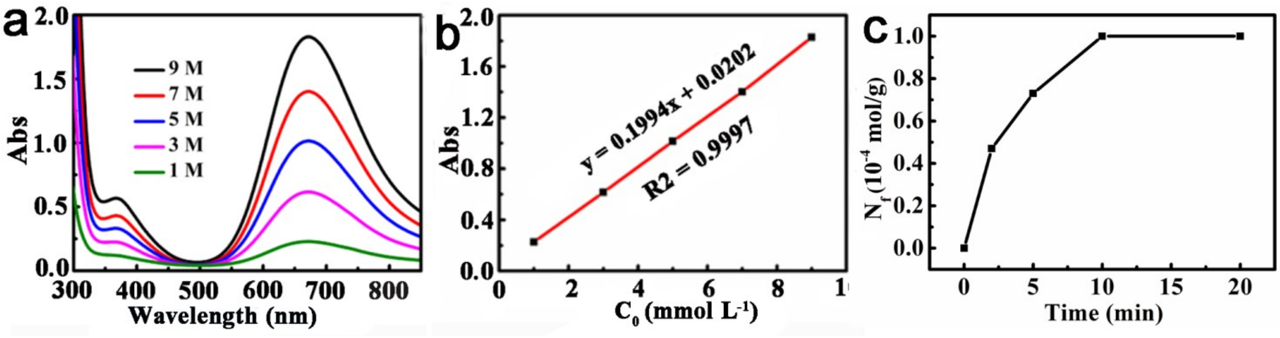

Fig. S9 Quantitative retention of copper ions by ACAT-Vitrimer. (a) UV-vis adsorption spectra of different concentrations of copper (**II**) acetate monohydrate/THF solution. (b) Standard absorption curve of copper (**II**) acetate monohydrate in THF. (c) Effect of contacting time on copper (**II**) ions adsorption capacity of ACAT-Vitrimer coated on silica gel.

7. Shape recovery of different samples

The shape memory effect of the non-vitrimer type epoxy with 1 mol% ACAT was checked. As shown in Fig. S11a, the temporarily spiraled (80ºC in oven) film could quickly recover its permanent shape when heated at 80ºC again. Moreover, the stretched film could also revert to its original shape via irradiation with light (0.22 W cm^{-2}) for just 10 s (Fig. S11b).

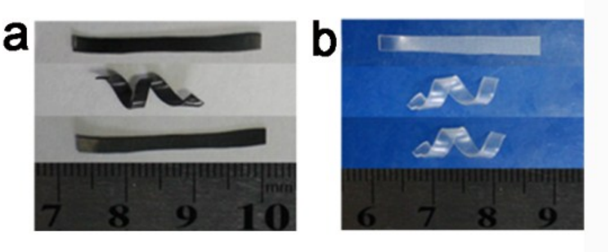

Fig. S10 The demonstration of photothermal effect induced shape recovery of ACAT-Vitrimer (a) and neat vitrimer(b).

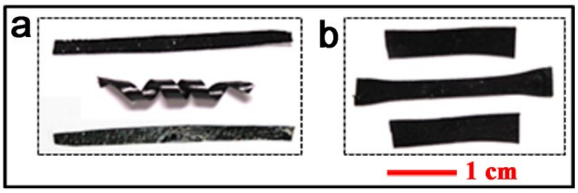

Fig. S11 The demonstration of shape recovery of non-vitrimer type epoxy with 1 mol% ACAT (with a thickness of about 0.2 mm). (a) Heat-induced shape recovery. (b) Light-triggered shape recovery.

8. Healing experiments by direct heating and light irradiation

To figure out whether the difference between direct heating and optical healing in our system is expansion, we designed another experiment. Three films were cut deeply with a knife for cracks with comparable size. One film was irradiated and consequentially photothermally heated in a uniform fashion (Fig. S12a-**I** and **II**), leading to a concerted expansion of the film as a whole (as would happen inside an oven, Fig. S12a-**III** and **IV**), while the third film was only irradiated with a narrow beam at the site of the inflicted defect, causing only this area to be heated significantly, and thus resulting in a local expansion that pushes or squeezes the borders of the defect more closer together, giving a much more efficient healing (Fig. S12b).

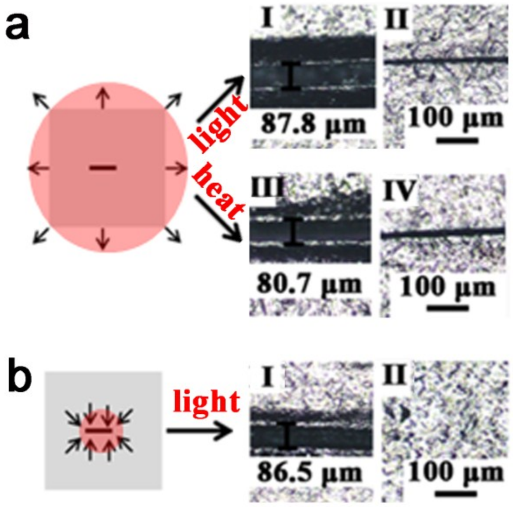

Fig. S12 Healing experiments of ACAT-Vitrimer. (a) Healing experiments conducted in a uniform fashion, where film **I** was irradiated by light (0.70 W cm^{-2}) for 20 s and film **III** was directly heated in oven at 180ºC for 10 min. (b) Healing experiment by irradiating (0.70 W cm^{-2}) with a narrow beam at the site of the inflicted defect for 20 s.

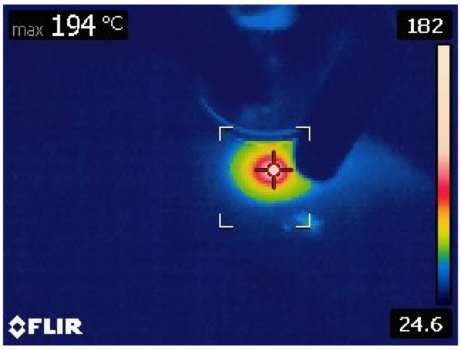

Fig. S13 Temperature of ACAT-Vitrimer around the cut when irradiated for 10s (light intensity: 0.70 W cm-2).

9. Investigation of the effect of the copper ion absorption on the properties of ACAT-Vitrimer

To investigate the effect of copper ion absorption on the properties of ACAT-Vitrimer, we prepared the following materials: ACAT-Vitrimer with 1 mol% ACAT before and after copper ion absorption and ACAT-Vitrimer with 10 mol% ACAT before and after the copper ion absorption (swelled in 3 mmol L^{-1} Cu (II)/THF solution for 20 min). The glass transition temperature T_g and transesterification temperature T_v of these samples were individually studied. As shown in Fig. S14 to S17, the copper ions absorption lowers T_g and the transesterification reaction rate of ACAT-Vitrimer. T_g on heating and the relaxation time τ* were summarized in Table 1.

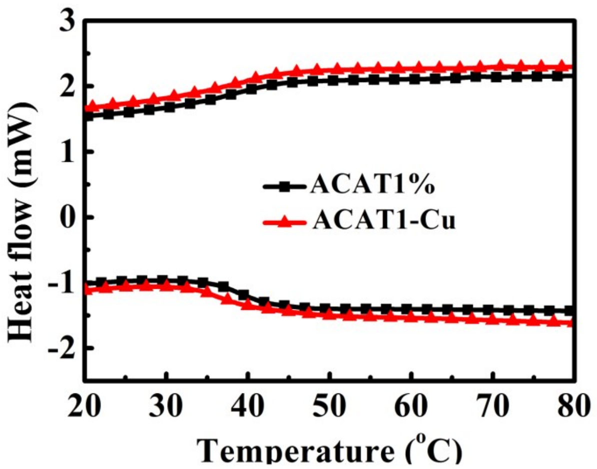

Fig. S14 DSC curves of ACAT-Vitrimer with 1 mol% ACAT before and after copper ions absorption.

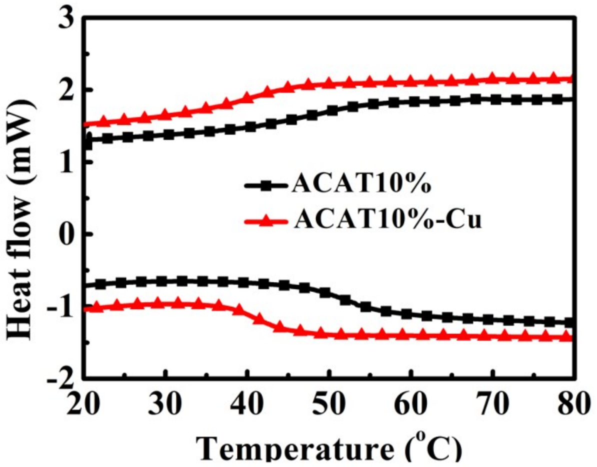

Fig. S15 DSC curves of ACAT-Vitrimer with 10 mol% ACAT before and after copper ion absorption.

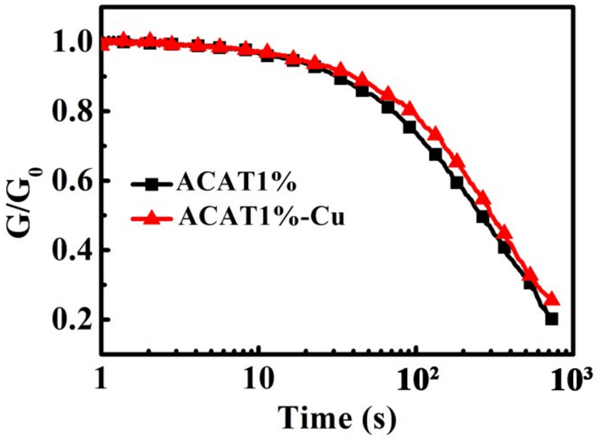

Fig. S16 Stress relaxation curves of ACAT-Vitrimer with 1 mol% ACAT before and after copper ion absorption.

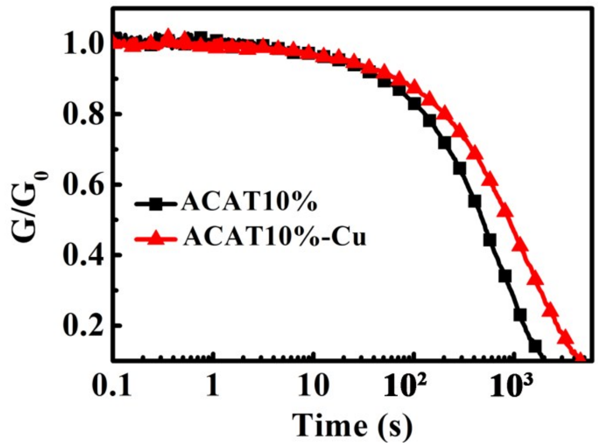

Fig. S17 Stress relaxation curves of ACAT-Vitrimer with 10 mol% ACAT before and after copper ion absorption.

Table 1 Summary of T_g on heating and the relaxation time τ* of different samples.

	ACAT 1mol%	ACAT 1mol% with Cu	ACAT 10mol%	ACAT 10mol% with Cu
Tg (°C)	40	36	53	42
τ* (s)	415	465	724	1458

10. PH response experiments

To check whether ACAT-Vitrimer could maintain the vitrimer property after treated with base, we conducted the recycling experiments. As shown in Fig. S18, pieces of the sample could still be combined together by hot-pressing but the recycling efficiency was not as excellent as the untreated one (Fig. S7e). The reason for this is that the transesterification catalyst (TBD) is a stronger base than TEA so, in contrast to ACAT, TBD would probably not be fully deprotonated. Thus some of the processability would lose (for ACAT still maintain its catalytic effect). We also studied the pH response of the non-vitrimer type epoxy with the same contents of ACAT. The sample was first swelled in tetrahydrofuran (THF) to reach swelling equilibrium then successively placed in acid (1 M p-toluene sulfonic acid, PTSA) and base (1 M triethylamine, TEA) (Fig. S19). From THF to acid, the length change was about 17% and from acid to base, the length change was about 18%. The experiments were repeated for several times, which reveals that the non-vitrimer type epoxy also has excellent pH responsive property.

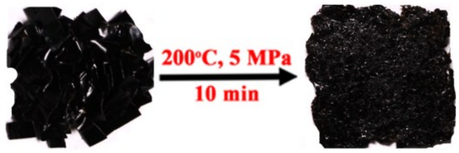

Fig. S18 Recycling experiments of ACAT-Vitrimer with 10 mol% ACAT after treated with acid then followed with base.

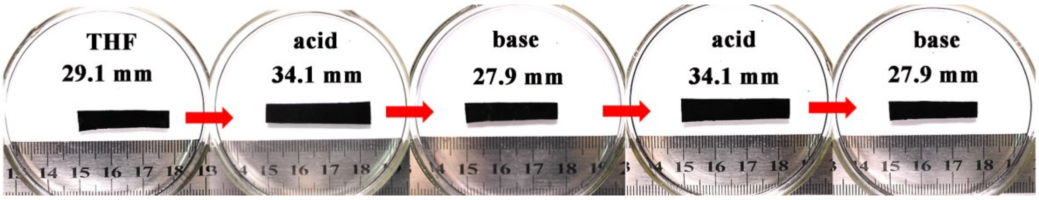

Fig. S19 Extension-contraction response of a non-vitrimer type epoxy film (the thickness before swelling is about 0.2 mm) with the pH changing, where the acid and base solutions were 1 M PTSA/THF solution and 1 M TEA/THF solution, respectively.

11. Swelling behaviors of the vitrimer with and without ACAT

The vitrimer films with and without ACAT were individually swelled in THF at room temperature. The equilibrium swelling ratios in length were 137% and 160%, respectively (Fig. S20).

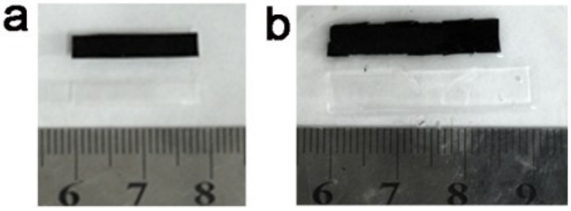

Fig. S20 Swelling behaviors of ACAT-Vitrimer and neat vitrimer. (a) Before swelling. (b) After swelling.

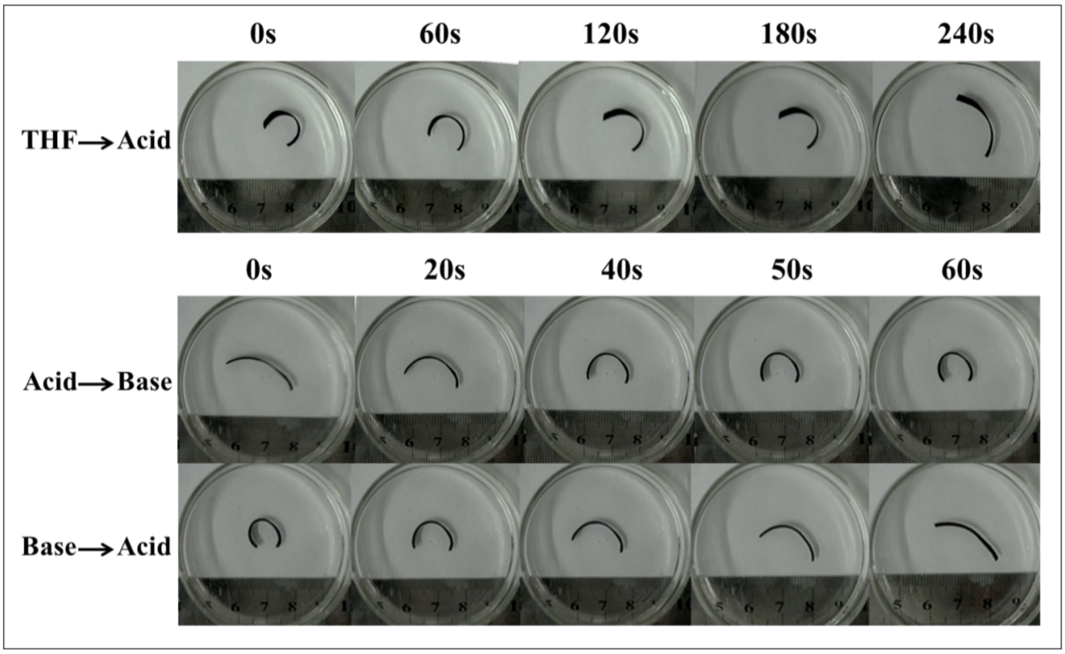

Fig. S21 Shape change of the bilayer in different pH, where the acid and base solutions were 1 M PTSA/THF solution and 1 M TEA/THF solution, respectively.

12. Films used in the multiple shape memory construct

The three films A, B and C were representative of ACAT-Vitrimer, ACAT-Vitrimer swelled in 1 mmol L^{-1} Cu (**II**)/THF solution for 20 min and ACAT-Vitrimer swelled in 3 mmol L^{-1} Cu (**II**)/THF solution for 20 min, respectively. The temperature of the three films at the different light intensities, in the cold environment about -20ºC (placed in the liquid nitrogen vapor as show in Fig. S22), was measured using an infrared thermal imager (Fig. S23).

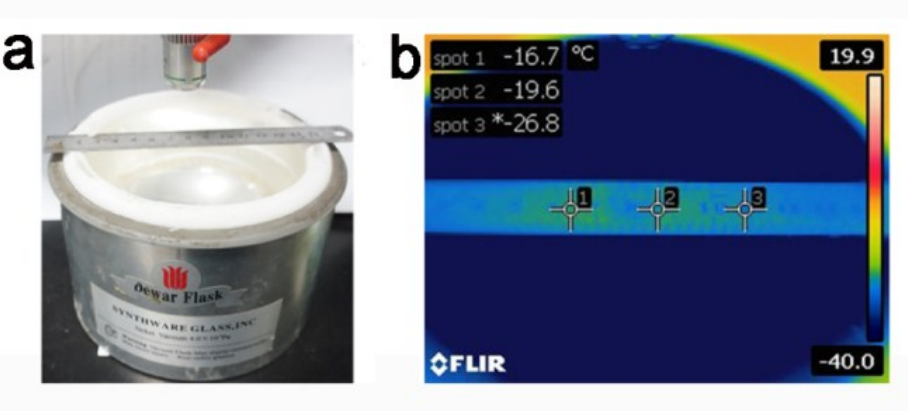

Fig. S22 (a) The equipment display for the multiple shape memory experiments in the liquid nitrogen vapor. (b) The temperature of the liquid nitrogen vapor measured using an infrared thermal imager.

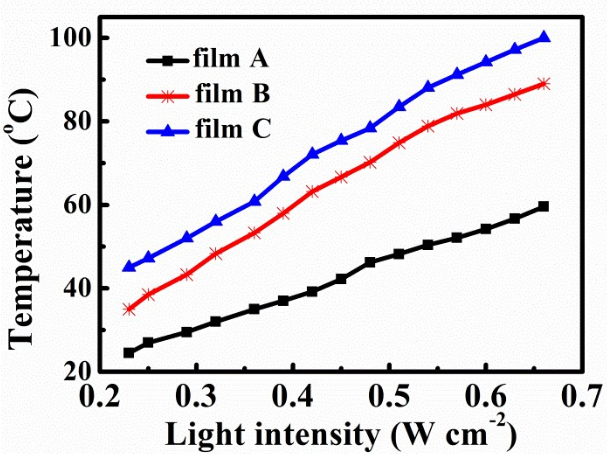

Fig. S23 The photothermal effect of the films A, B and C (representative of ACAT-Vitrimer, ACAT-Vitrimer swelled in 1 mmol L^{-1} Cu (**II**)/THF solution for 20 min and ACAT-Vitrimer swelled in 3 mmol L^{-1} Cu (**II**)/THF solution for 20 min) in the cold environment about -20ºC.

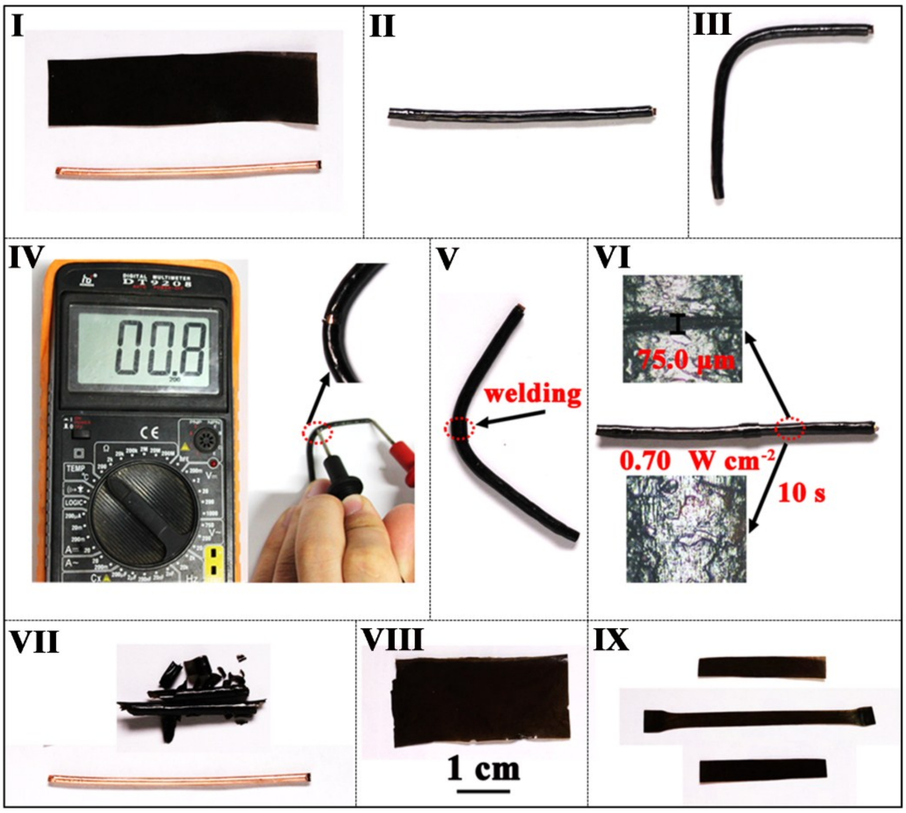

Fig. S24 A demonstration of the ACAT-Vitrimer being used as the insulation coat of a copper wire. (I) The square ACAT-Vitrimer film (with a thickness of about 0.2 mm) and a copper wire before wrapping. (II) The wrapped wire made by heat-welding ACAT-Vitrimer at 200 ºC for 10 min in oven. (III) A new permanent shape of the wrapped wire. (IV) Measuring the resistance between the crack and the end of the wire using a digital mutilmeter. The crack has a width of about 2 mm. (V) The mended wire. To insure an excellent mending, we twined a small piece of film above the crack then irradiated with light (0.70 W cm^{-2}) for 1 min. (VI) A microscale crack on the straightened wire was optically healed in suit (0.70 W cm^{-2}) for 10 s. (VII) The ACAT-Vitrimer was peeled off. (VIII) The recycled ACAT-Vitrimer film made by hot-pressing under a pressure of 5 MPa for 10 min at 200ºC using the ACAT-Vitrimer peeled off from the wire. (IX) The shape memory effect of the recycled film.
